# Interpretable Machine Learning for Inpatient COVID-19 Mortality Risk Assessments: Diabetes Mellitus Exclusive Interplay

**DOI:** 10.3390/s22228757

**Published:** 2022-11-12

**Authors:** Heydar Khadem, Hoda Nemat, Jackie Elliott, Mohammed Benaissa

**Affiliations:** 1 Department of Electronic and Electrical Engineering, University of Sheffield, Sheffield S10 2TN, UK; 2 Department of Oncology and Metabolism, University of Sheffield, Sheffield S10 2TN, UK; 3 Teaching Hospitals, Diabetes and Endocrine Centre, Northern General Hospital, Sheffield S5 7AU, UK

**Keywords:** COVID-19, diabetes mellitus, machine learning, SHAP

## Abstract

People with diabetes mellitus (DM) are at elevated risk of in-hospital mortality from coronavirus disease-2019 (COVID-19). This vulnerability has spurred efforts to pinpoint distinctive characteristics of COVID-19 patients with DM. In this context, the present article develops ML models equipped with interpretation modules for inpatient mortality risk assessments of COVID-19 patients with DM. To this end, a cohort of 156 hospitalised COVID-19 patients with pre-existing DM is studied. For creating risk assessment platforms, this work explores a pool of historical, on-admission, and during-admission data that are DM-related or, according to preliminary investigations, are exclusively attributed to the COVID-19 susceptibility of DM patients. First, a set of careful pre-modelling steps are executed on the clinical data, including cleaning, pre-processing, subdivision, and feature elimination. Subsequently, standard machine learning (ML) modelling analysis is performed on the cured data. Initially, a classifier is tasked with forecasting COVID-19 fatality from selected features. The model undergoes thorough evaluation analysis. The results achieved substantiate the efficacy of the undertaken data curation and modelling steps. Afterwards, SHapley Additive exPlanations (SHAP) technique is assigned to interpret the generated mortality risk prediction model by rating the predictors’ global and local influence on the model’s outputs. These interpretations advance the comprehensibility of the analysis by explaining the formation of outcomes and, in this way, foster the adoption of the proposed methodologies. Next, a clustering algorithm demarcates patients into four separate groups based on their SHAP values, providing a practical risk stratification method. Finally, a re-evaluation analysis is performed to verify the robustness of the proposed framework.

## 1. Introduction

Shortly after the outbreak of coronavirus disease-2019 (COVID-19), pre-existing diabetes mellitus (DM) was recognised as a risk factor for the new disease [1,2]. Subsequently, extensive research has been underway to study this vulnerability [3]. For instance, adopting logistic regression (LR) analysis, Sourij et al. investigated the prognostic prediction in hospitalised COVID-19 patients with DM. They also offered a simple yet effective score for forecasting the risk of fatal outcomes from age and on-admission values of arterial occlusive disease, c-reactive protein (CRP), estimated glomerular filtration rate, and aspartate aminotransferase [4].

DM comorbidity was later declared a leading cause of in-hospital COVID-19 mortality in some studies [5]. As an example, in a uni-centre retrospective study, Ciardullo et al. deployed LR to perform death prediction analysis for 373 hospitalised COVID-19 patients with DM from diabetes status, comorbid conditions, and laboratory data. Based on the results achieved, the authors affirmed DM as an independent culprit for in-hospital COVID-19 mortality [6].

Although the COVID-19 susceptibility of DM patients has been well documented, explaining this vulnerability remains a challenge [3,7]. The use of explainable machine learning (ML) is one strategy to contribute to addressing this challenge [8,9,10,11].

In general, ML algorithms carry excellent potency in discovering intricate correlated interactions [8,12]. These tools have found practical implementation in COVID-19 research. As a representative, using machine learning techniques, Kar et ll. studied 63 clinical and laboratory factors in relation to 1393 subjects hospitalised for COVID-19 to forecast the probability of mortality at 7 and 28 days. As a result, they generated an effective bespoke death risk score [13].

Integrating underlying ML pipelines with model interpretation frameworks promotes the transparency of the analysis, offsetting the block-box nature of plain ML algorithms [14,15,16]. SHapley Additive exPlanations (SHAP) is an exemplar of elaborate ML explainability techniques [17,18]. SHAP employs the classical notion of Shapley values from cooperative game theory to measure the contribution of input data in forming a given output by the model [19]. The measured SHAP values for a particular input feature indicate the deviation from the average prediction when conditioned on that feature [18].

SHAP analysis has seen promising applications in COVID-19 risk assessment research [19,20,21]. For example, Pan et al. designed ML models dressed with SHAP analysis for COVID-19 prognosis assessment in individuals hospitalised in intensive care units [20].

In a recent publication, we developed machine learning pipelines incorporated with interpretation components for mortality risk prediction and stratification in hospitalised COVID-19 patients with and without DM in parallel [22]. For this purpose, a set of features collected at the point of hospital admission for both groups were investigated. Consequently, the generated risk assessment models possessed potential application to triage systems for both groups and enabled inter-cohort comparative analysis. In the original dataset, though, there existed a pool of historical, on-admission, and inpatient variables collated only for the DM group. These features were either DM-relevant, or the primary investigations did not persuade the clinical data acquisition team to collate for the non-DM cohort. Due to the significance of the topic and the value of further knowledge discovery in this field, the current sequel study is conducted to create risk assessment platforms comparable to those in the earlier study for the same DM cohort by scrutinising only the abovementioned DM-exclusive data opted out of the former investigation.

First, the clinical data are cleansed and prepared for formal ML modelling analysis. After that, an ML model is constructed for inpatient fatality risk assessments. In-depth evaluation and interpretation analyses are performed on the model, and the results obtained are discussed in detail. This follow-on work initially recruits similar main methods contrived in the prior paper, focusing on new findings, discussions, and applications. This homogeneity facilitates the analogical study of the two relevant articles. Next, some compartments in the primary skeleton of the pipelines are replaced with new units, and the investigations are re-conducted. This complementary analysis further inspects the robustness of the infrastructure utilised in the two studies by a side-by-side comparison of the new and old outcomes.

The remainder of the paper is organised as follows. The clinical data utilised in this work are outlined in Section 2. In Section 3, data pre-treatment steps undertaken before the conventional ML modelling analysis are explained. Section 4 describes the primary methodologies implemented for mortality risk assessments. The results achieved and the associated discussion is represented in Section 5. Section 6 reports further stability investigations on the proposed work frames. Finally, Section 7 summarises and concludes the work.

## 2. Material

The source of the clinical data used in this paper is the dataset primarily described in [23]. The present research explores demographic, clinical, and laboratory data from 156 individuals in the main dataset with confirmed COVID-19 and DM comorbidity. All these participants were admitted to Sheffield Teaching Hospitals (Sheffield, UK) between 29 February 2020 and 1 May 2020. Of the 156 patients, 103 survived, and 51 died due to COVID-19; the other three died due to causes other than COVID-19, according to their death certificates. Table 1 and Table 2 summarise the statistical characteristics of the data used in this work. Table 1 includes categorical variables’ information encompassing the name of categories and the number of recorded data in each category. In Table 2, the mean and standard deviation (SD) of numerical variables are presented alongside the frequency of records for each feature.

## 3. Data Curation

The following four pre-treatment stages are undertaken to prepare the data for the ensuing ML modelling analysis.

### 3.1. Cleaning

In the first data-cleaning step, tainted entities and features are excluded from the rest of the analysis. First, the three individuals with reported non-COVID-19 mortality reasons (as discussed in Section 2) are omitted from the rest of the analysis. Next, features and participants with a high missingness rate are discarded. For this purpose, an inclusion criteria of having a missingness rate of no more than 50% is determined for both features and individuals [24]. Initially, attributes with missing rates larger than the 50% threshold are discarded. Next, the same criterion is applied to data contributors. As a result, the following features are omitted from the rest of the analysis: FI-HbA1c, LV-ALT, HV-ALT, LV-Procalcitonin, HV-Procalcitonin, LV-Ferritin, HV-Ferritin, OA-Troponin, LV-Troponin, HV-Troponin, LAYBA-UACR, LAYBA-Vitamin D, LV-PT, HV-PT, LV-APTT, HV-APTT, LV-Fibrinogen, HV-Fibrinogen, LV-D-dimer, HV-D-dimer, and FI-Ketones. However, no further individual is obviated from the rest of the analysis, as no one holds more than 50% missingness after discarding the abovementioned high-missing rate features.

### 3.2. Subsetting

After the cleaning phase, data that have met the inclusion criteria and qualified for the subsequent analysis are subdivided into training and testing sets as per the requirements of upcoming supervised ML analysis. For data subsetting, 70% of the cases are allocated as the training set and 30% as the testing set. Stratified random sampling carries out the train-test split process to take into account the distribution of classes. All model training and hyperparameter tuning operations are undertaken on training sets only, with testing sets remaining unseen for evaluation and model interpretation analysis.

### 3.3. Pre-Processing

Three pre-processing steps are conducted to render the data more suitable for ML analysis: outlier treatment, missing value imputation, and feature transformation.

Initially, leveraging the winsorisation technique, we shift the numerical variables placed outside the 5th to 95th percentile to the corresponding boundary. This confinement pre-empts extreme values of skewing the results.

Next, the missing values for numerical features are treated using the k-nearest neighbour data imputation technique [25], configuring the number of neighbours as five. Exploring all non-missing features, the algorithm selects five data entities from the training set with the most congruency with a given data contributor. Then the average of these akin points is used to interpolate the missing values of the given data instance. For categorical variables, the most repeated value is used to fill in the missing values.

Lastly, features are transformed into a more digestible form for ML algorithms. Categorical attributes are converted to numerical form using the binary encoding technique. The numerical features are standardised. The mean of the training set is subtracted from each feature, and then the results are scaled to unit variance by dividing them by the standard deviation of the training set.

### 3.4. Feature Elimination

After the data curation steps, a voting feature selection is performed on the pre-processed data to reduce the input size and help preclude the occurrence of a dimensionality curse. First, regular LR, gradient boosting (GB), and AdaBoost (AB) models, which all have already succeeded in applications to COVID-19 research, are fine-tuned. To this end, the random search approach is used to select the hyperparameter values delivering the highest five-fold cross-validation accuracy on the training set. The outcomes of hyperparameter tuning are given in Table A1, Appendix. Next, the recursive feature elimination technique is enfolded around each model, forming a voting system. Each voting system then shortlists 15 features (approximately one-tenth the number of data points, a common practice in ML modelling) by investigating training data only. The features picked by at least two voting systems are then used as predictors to generate the final mortality risk prediction model. The shortlisted features encompass LAYBA-NEUT, HV-NEUT, LaV-LYM, LAYBA-MN, OA-MN, LV-Platelets, HV-Platelets, LV-CRP, LAYBA-PT, FI-BGL, FI-RR, FI-FiO2, and HR-O2.

## 4. Modelling

This section develops explainable ML models for mortality risk assessment analysis from the selected features. Prior to representing model implementations, providing a brief description of SHAP theory and calculations is of use.

### 4.1. Preliminary

As a game-theoric model agnostic method, SHAP simulates the formation of outputs by an ML model as a game. In this gamification process, the input features have the role of involved players. Subsequently, the payoff for each player in the game is calculated as Equation (1) [18], based on the principles of Shapley value [19]. On elucidating the formula, the SHAP value of a particular feature for a given individual is calculated by integrating the payoff shares for the feature in all possible coalitions with other variables. The payoff share of the feature in each coalition is determined by calculating the difference between the whole payoff of the coalition with and without the given feature included and then dividing the outcome between the members of the coalition equally.

(1) $$SHAP_{x}\left(f\right)=\underset{F:f\in F}{\sum }{\left(\left|F\right|\times \left(\begin{array}{c} N\\ \left|F\right| \end{array}\right)\right)}^{-1}\times \left({\hat{x}}_{F}-{\hat{x}}_{F\backslash f}\right)$$

*f*: a given feature; *x*: a given data point; $SHAP_{x}\left(f\right)$: SHAP value of variable *f* for *x* (the payoff of feature *f* in the designed game); *F*: all permutations of feature with *f* included; |F|: the size of *F* (number of features in *F*); *N*: the whole number of features in the models; ${\hat{x}}_{F}$: the model’s output for *x* using the feature subset *F*; ${\hat{x}}_{F\backslash f}$: the model’s output for *x* from the feature subset *F* excluding *f*.

### 4.2. Mortality Risk Prediction

The first risk assessment analysis is to forecast in-hospital COVID-19 mortality from selected features. For this purpose, a random forest (RF) classifier, which has proven its capability in COVID-19 risk assessment research [26], is fine-tuned using the same approach explained in Section 3.4. The results of this optimisation analysis are presented in Table A1, Appendix. The fine-tuned RF classifier is then trained on the entire training set to predict inpatient death due to COVID-19. Following that, the generated model undergoes careful evaluation analysis employing four widely used metrics: accuracy, area under the receiver operating characteristic curve (AUC), sensitivity, and specificity. After evaluating the mortality risk prediction model, SHAP is leveraged to interpret the model globally and locally.

### 4.3. Mortality Risk Stratification

**Figure 1:**
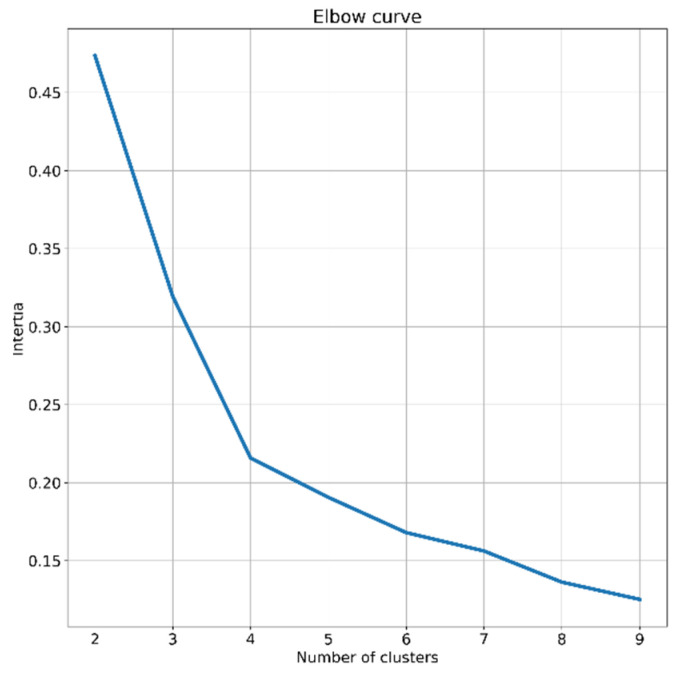
A schematic of elbow analysis operated to decide the number of clusters.

The second risk assessment analysis is to stratify the in-hospital mortality risk of patients. In order to do so, SHAP clustering [27], an extension of SHAP analysis, is deployed. The k-means [28], an algorithm used in previous COVID-19 research [29,30], is employed on SHAP values to search for meaningful clusters of individuals. The algorithm clusters the subjects into an optimised number of groups [22] with identical variance by optimising a criterion known as inertia [28]. For deciding the number of clusters, the heuristic elbow method is employed. Values of 1 to 9 are explored, and the one resulting in an elbow point, based on inertia values achieved, is chosen [31]. According to the outcome of elbow analysis shown in Figure 1, four is determined as the number of clusters, as the diagram has the sharpest break point for this value.

## 5. Results and Discussion

This section presents the results of model evaluation and interpretation analysis alongside the corresponding discussion. First, the outcomes of mortality risk prediction analysis are given and then those of mortality risk stratification analysis.

### 5.1. Mortality Risk Prediction

#### 5.1.1. Evaluation

The generated mortality risk prediction model provides these evaluation results across the testing set: 97% accuracy, 78% AUC, 78% sensitivity, and 80% specificity. Such practical evaluation results support the overall effectiveness of the implemented methodologies, including the feature selection, and hyperparameter tuning processes coupled with the final RF classifier. These dependable outcomes also backend the following SHAP-based analysis.

#### 5.1.2. Global Interpretations

The next results to present for the mortality risk prediction model are the outcome of the interpretation analysis. In this regard, first, the results of global interpretation analysis are reported, followed by those of local interpretation analysis.

**Figure 2:**
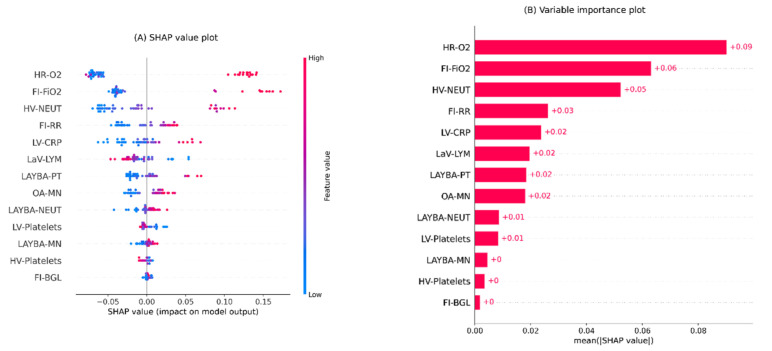
Global interpretation plots for the developed inpatient COVID-19 mortality prediction model. (**A**) Bee swarm SHAP values plot, (**B**) SHAP summary importance plot. The bee swarm plot shows all SHAP values in accord with predictors values. The summary plot presents predictors in descending order based on their overall importance on the model’s outcomes derived from mean absolute SHAP values. Note. BGL: blood glucose level; CRP: c-reactive protein; FI: first inpatient; FiO2: fraction of inspired oxygen; HR: highest requirement; HV: highest value; LaV: last value; LAYBA: latest available within one year before admission; LV: lowest value; LYM: lymphocytes; MN: monocytes; NEUT: neutrophils; OA: on admission; O2: oxygen; PT: prothrombin time; RR: respiratory rate; SHAP: SHapley Additive exPlanations.

Figure 2 illustrates the results of global interpretations for the generated mortality risk prediction model in two plots. Both plots represent the features in descending order as per their overall influence on the model’s outcomes.

The bee swarm plot in Figure 2A shows SHAP values and their relative association with predictions of death. Each point on the scheme represents a feature value from the testing set. The values of features are colour-coded from blue to red, encoding low to high values. A positive SHAP value for each point denotes the adverse effect of the feature, viz, its contribution level to a higher risk of death. In contrast, a negative SHAP value indicates the protective effect of the relevant feature, i.e., decreasing the risk of death.

The bar chart in Figure 2B is the variable importance plot for the developed mortality risk prediction model. The plot summarises the features’ overall impacts on the model outputs according to their mean absolute SHAP values, represented by the length of the bars.

According to Figure 2A, the predictors positively associated with mortality risk predictions are HR-O2, FI-FiO2, HV-NEUT, FI-RR, LV-CRP, LAYBA-PT, OA-MN, LAYBA-NEUT, and LAYBA-MN. By comparing the positive and negative SHAP values of these variables, it is noticeable that HR-O2, FI-FiO2, HV-NEUT, and LAYBA-PT show greater adverse than protective effects. In other words, the sinister roles of higher values for these features are relatively more significant than the protective roles of lower values. On the other hand, with similar explanations, it can be inferred that FI-RR carries a stronger protective than adverse power. The rest of the aforementioned variables have comparable protective and adversarial influences. On the other side, based on the plot, it can also be seen that the modalities negatively associated with the prediction of death comprise LaV-LYM, HV-Platelets, and LV-Platelets. Overall, the first two variables possess more substantial sinister impacts (in lower values) than protective impacts (in higher values), whereas the last one holds stronger protective effects (in higher values) than sinister effects (in lower values).

Furthermore, based on Figure 2A, one noteworthy inference is that a more influential feature does not necessarily have stronger adverse and protective power at once. To exemplify, notwithstanding the greater overall importance of HR-O2 over FI-FiO2, the latter possesses a more substantial adverse impact than the former on average. This deduction is formed based on predominantly bigger positive SHAP values for FI-FiO2 compared to HR-O2.

Additionally, according to the plots in Figure 2, HR-O2, FI-FiO2, and HV-NEUT form the top three influential features with considerably higher impacts than others. Therefore, undesired measures for these features may be an indicator of high death risk. These findings underscore the importance of careful inpatient surveillance and the monitoring of peak values of oxygen requirement and NEUT, along with the imperative role of immediate inspection of FI-FiO2 after admission for COVID-19 patients with DM.

Another notable point is that two features from the patients’ historical profiles, LAYBA-PT and LAYBA-NEUT, have shown considerable mortality predictivity power even in the presence of many on-admission and during-admission data. This observation stresses the potential utility of accessing and considering the history profile of COVID-19 patients with DM, and specifies two features as candidates with high priority for consideration in this respect.

#### 5.1.3. Local Interpretations

**Figure 3:**
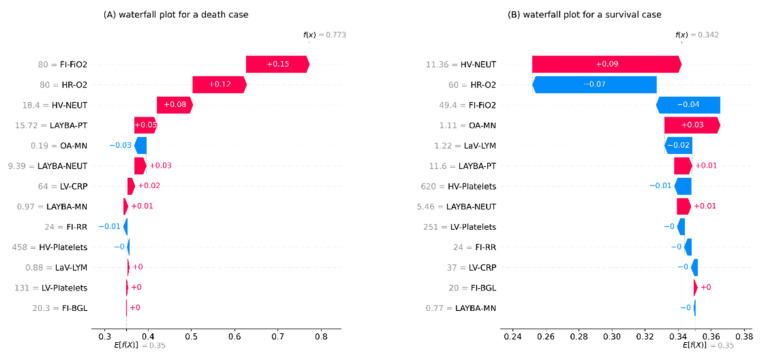
The local interpretation plots for the developed inpatient COVID-19 mortality prediction model. (**A**) an example of patients with survival as the outcome of admission, (**B**) an example of patients with death as the outcome of admission. The plots start from the bottom with a predefined prediction for the risk of death equal to the average death rate in the training set. Next, the arrows with an ascending order show how each feature has contributed to the formation of a final prediction specified for the given data instance shown at the top of the plot. Note. BGL: blood glucose level; CRP: c-reactive protein; FI: first inpatient; FiO2: fraction of inspired oxygen; HR: highest requirement; HV: highest value; LaV: last value; LAYBA: latest available within one year before admission; LV: lowest value; LYM: lymphocytes; MN: monocytes; NEUT: neutrophils; OA: on admission; O2: oxygen; PT: prothrombin time; RR: respiratory rate; SHAP: SHapley Additive exPlanations.

The next outcome to be presented entails the results of local interpretation analysis for the mortality risk prediction model. In this respect, Figure 3 shows the waterfall plots for two randomly selected examples of data entities, one from individuals with death and the other from those with survival as the outcomes of their admissions. These plots start at the base from E[*f*(*x*)], representing the average risk of death according to the training set. Next, each arrow illustrates the influence of a feature, i.e., the feature’s SHAP value, towards forming the specific prediction for the given entry. The positive associations of the given feature with increased mortality risk prediction are exhibited by red rightward arrows and the negative associations with blue leftward arrows. Finally, at the top of the plot, the model’s output for the given sample is represented by *f*(*x*). It merits mentioning that each arrow’s length denotes the level of impact from its relevant feature, i.e., absolute SHAP value. Moreover, the arrows are displayed in ascending order from the bottom to the top of the plots according to their size.

One immediate recognition from both plots in Figure 3 is that the grade and order for the features’ impacts on local interpretations are different from global interpretations. This evidence shows how local interpretations can evolve the transparency of the analysis by explaining the formation of each specific outcome through localising and contrasting the effect of the components, as opposed to giving a generic explanation based on all outcomes.

For the death instance represented in Figure 3A, relatively high values for features FI-FiO2, HR-O2, HV-NEUT, and LAYBA-PT have been the most effective predictors of a fatal outcome. In this regard, it is worth remarking that, in line with the aforementioned discussion, FI-FiO2 had a more adverse impact than HR-O2, the most influential feature overall.

For the survival case reported in Figure 3B, features with protective impacts are HR-O2, FI-FiO2, LaV-LYM, and HV-Platelets. It is worth highlighting that this case has received a non-fatal outcome prediction, whilst its influential feature, HV-NEUT, shows an adverse impact. Moreover, for this data instance, HR-O2 and Fi-FO2 both deliver a protective effect, with the former’s being stronger. This perception is also in line with the overall higher protective influence of HR-O2, as discussed before.

### 5.2. Mortality Risk Stratification

Table 3 outlines the results of the SHAP clustering analysis, including the distribution of patients in the generated clusters, the rate of mortality outcome in each cluster, and a summary of the statistical characteristics of predictors within clusters. Based on the table, it can be apprehended that the clustering approach has made an appropriate risk stratification system by forming four categories with disparate characteristics.

In terms of mortality rates, cluster 1 poses a zero mortality rate, cluster 2 has a moderate mortality rate, and clusters 3 and 4 have relatively high mortality rates. Further distinctive patterns can be found based on the feature distributions within clusters, specifically for the more critical variables. For example, a pronounced discriminator between clusters 1 and 2 compared to clusters 3 and 4 is that the first two have an average HR-O2 considerably lower than the other two. Additionally, comparing clusters 1 and 2, a prominent discrepancy is that the former, in general, includes patients with more desired values for HV-NEUT and LV-CRP. One more pattern to mention is that a significant distinguisher between clusters 3 and 4 is the relatively higher average values for FI-FiO2 and LV-CRP in cluster 3.

## 6. Complementary Analysis

This section presents some extra analysis embarked on for robustness assessments. The following set of amendments is applied to the compartments of the proposed learning environment. The data are reshuffled in their entirety, and a new round of 30–70 stratified random samplings is performed to reallocate training and testing sets. In addition, missing values are interpolated using a different technique by applying the iterative imputation. In order to do so, a Bayesian ridge regressor is set as the estimator for the numerical variables and an RF classifier for the categorical variables. In addition, the mortality risk prediction modelling is performed again using a support vector classifier (SVC). The SVC is fine-tuned using the random search approach described in Section 3.4, and the results are presented in Table A1, Appendix. Following these updates, the evaluation, interpretation, and clustering analyses are re-conducted. The new results are presented concisely below, and the consistency of the proposed core workflow in producing practical outcomes in line with previously discussed findings is inspected.

The features selected in the updated analysis are BGV-Na, BGV-Cl, LV-NEUT, HV-NEUT, LaV-LYM, LV-Platelets, HV-Platelets, LV-Albumin, LV-CRP, Preadmission-SBP, DM duration, FI-RR, FI-FiO2, and HR-O2. In comparison, the top six most important features, according to the previous analysis (HR-O2, FI-FiO2, HV-NEUT, FI-RR, LV-CRP, and LaV-LYM), are all shortlisted in the renewed analysis as well.

Furthermore, the updated fatality risk prediction model yields these new evaluation outcomes over the testing set: 87% accuracy, 92% AUC, 72% sensitivity, and 74% specificity. Similar to the primary analysis, these results are practical, with an outcome of more than 70% for every metric.

**Figure 4:**
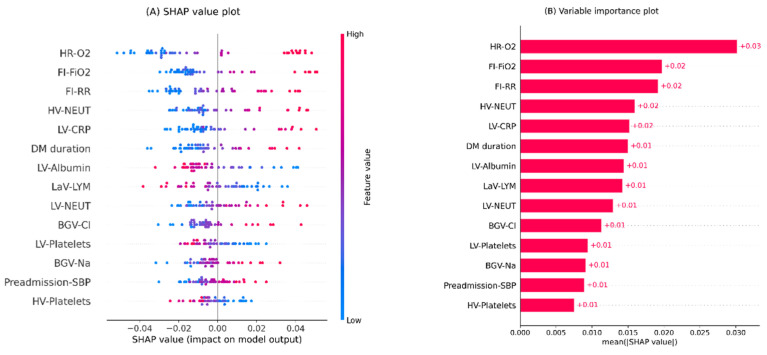
Global interpretation plots for the updated inpatient COVID-19 mortality prediction model. (**A**) Bee swarm SHAP values plot, (**B**) SHAP summary importance plot. The bee swarm plot shows all SHAP values in accord with predictors values. The summary plot presents predictors in descending order based on their overall importance on the model’s outcomes derived from mean absolute SHAP values. Note. BGV: blood gas value; Cl: chloride; CRP: c-reactive protein; DM: diabetes mellitus; FI: first inpatient; FiO2: fraction of inspired oxygen; HR: highest requirement; HV: highest value; LaV: last value; LV: lowest value; LYM: lymphocytes; Na: sodium; NEUT: neutrophils; O2: oxygen; RR: respiratory rate; SBP: systolic blood pressure; SHAP: SHapley Additive exPlanations.

Moreover, Figure 4 illustrates the global interpretation plots for the renewed models. According to the plots, HR-O2 and FI-FiO2 are the first and second most important features, similar to the primary analysis. In addition, the top five ranks are occupied by the same features in the original and renewed analysis.

Furthermore, Table 4 shows the results of the new SHAP clustering analysis. For brevity, only the top four important features are shown in the table. As can be seen, similar to the primary analysis, the new SHAP clustering analysis successfully groups patients into four distinguishable categories.

All in all, based on the discussion above, there is a significant agreement between the results of both the updated and the original analysis. This alignment promises the robustness of the core interpretable ML workflow proposed for mortality risk assessment in COVID-19 patients.

## 7. Conclusions

Inpatient COVID-19 mortality risk assessment specifically designed for patients with pre-existing DM was performed in this work. This goal was achieved by investigating a set of clinical features exclusively pertaining to DM and COVID-19 interplay for 156 individuals. Initially, the clinical data of the studied subjects were carefully pre-treated for the subsequent standard ML modelling analysis. After that, a mortality risk prediction model was created, exercising established ML pipelines. Evaluation analysis was then performed on the generated model. The results underpinned the effectiveness of the data treatment and modelling analysis. Afterwards, the generated model was interpreted globally and locally using SHAP. These interpretations help extend the transparency of the analysis. Next, a mortality risk stratification system was developed upon the outcomes of the SHAP analysis. Finally, an extra analysis was performed to further examine the stability of the core pipelines, where the outcomes corroborated this. The analysis reported in this work can be applied to online surveillance of hospitalised patients. The findings suggest some critical features to be reviewed more carefully in this monitoring process. To further expand upon this area of knowledge, future work could include more rigorous scrutiny of SHAP clustering results by devising a nested model interpretation mechanism.

## Figures and Tables

**Table 1:** A summary of properties of the categorical clinical data used in this article. For each feature, the categories’ names and the number of patients with recorded data in each category are given.

Feature	Category	Count	Feature	Category	Count
CKD	Yes	131	PC-Dizziness	Yes	7
	No	21		No	145
Radiology-RPLD	Yes	12	PC-Headache	Yes	5
	No	132		No	147
Radiology-Consolidation on report	Yes	99	PC-Hyperglycaemia	Yes	12
	No	46		No	140
Radiology-Worsening consolidation	Yes	34	DM type	Type 1	12
	No	25		Type 2	140
Radiology-CT chest or CTPA	Yes	5	DM complications	Yes	127
	No	143		No	26
Radiology-PE	Yes	1	PVD	Yes	47
	No	5		No	105
Diabetes autoantibodies	Yes	1	Peripheral neuropathy	Yes	67
	No	152		No	84
PC-Fever	Yes	64	Background retinopathy	Yes	73
	No	88		No	64
PC-Cough	Yes	81	Preproliferative retinopathy	Yes	15
	No	72		No	122
PC-SOB	Yes	66	Proliferative retinopathy	Yes	17
	No	86		No	120
PC-Chest pain	Yes	11	Previous foot ulcer	Yes	28
	No	141		No	124
PC-Abdominal pain	Yes	8	Active foot ulceration	Yes	8
	No	144		No	144
PC-Diarrhoea	Yes	21	VRII treatment during admission	Yes	23
	No	131		No	129
PC-Myalgia	Yes	16	DNAR	Yes	109
	No	136		No	41

Note. CKD: chronic kidney disease; CT: computed tomography; CTPA: computed tomography pulmonary angiogram; DNAR: do not attempt resuscitation; PC: presenting complaint; PE: pulmonary embolism; PVD: peripheral vascular disease; RPLD: reported pre-existing lung disease; SOB: shortness of breath; VRII: variable rate intravenous insulin infusion.

**Table 2:** A summary of properties of the numerical clinical data used in this article. For each feature, mean and standard deviation, together with the number of patients that a value is recorded for the feature, are given.

Feature	Mean ± SD	Count	Feature	Mean ± SD	Count
BGV-pH (mmol/L)	7.39 ± 0.09	80	LV-ALPO4 (g/L)	87.27 ± 49.08	100
BGV-HCO3 (mmol/L)	22.43 ± 4.33	79	HV-ALPO4 (g/L)	134.35 ± 87.84	100
BGV-Lactate (mmol/L)	2.09 ± 1.71	80	LAYBA-Albumin (g/L)	39.82 ± 5.17	149
BGV-Na (mmol/L)	134.83 ± 5.45	80	LV-Albumin (g/L)	30.15 ± 4.91	104
BGV-K (mmol/L)	4.13 ± 0.86	80	HV-Albumin (g/L)	37.4 ± 4.2	104
BGV-Cl (mmol/L)	99.65 ± 10.91	80	LV-CRP (mg/dL)	49.37 ± 57.93	133
BGV-Anion Gap (mmol/L)	16.86 ± 10.44	79	HV-CRP (mg/dL)	165.89 ± 124.04	133
LAYBA-HbA1c (mmol/mol)	61.34 ± 17	144	LV-Procalcitonin (µg/L)	0.58 ± 1.04	68
FI-HbA1c (mmol/mol)	68.01 ± 19.52	74	HV-Procalcitonin (µg/L)	3.04 ± 11.98	68
LAYBA-Hb (g/L)	123.9 ± 20.89	146	LV-Ferritin (µg/L)	1019.87 ± 1363.47	45
LV-Hb (g/L)	106.5 ± 20.22	140	HV-Ferritin (µg/L)	1596.16 ± 2861.80	45
HV-Hb (g/L)	125.91 ± 18.29	140	OA-Troponin (ng/L)	49.07 ± 70.64	25
LAYBA-WCC (g/L)	8.09 ± 2.95	147	LV-Troponin (ng/L)	90.75 ± 224.18	32
LV-WCC (g/L)	6.03 ± 2.84	140	HV-Troponin (ng/L)	108.94 ± 237.01	32
HV-WCC (g/L)	11.01 ± 5.69	140	LAYBA-UACR	35.07 ± 76.42	62
LAYBA- NEUT (109/L)	5.35 ± 2.47	147	LAYBA-Vitamin D (ng/mL)	44.25 ± 25	44
LV- NEUT (109/L)	4.24 ± 2.43	140	LAYBA-PT (s)	12.26 ± 4.12	92
HV- NEUT (109/L)	8.72 ± 5.05	140	LV-PT (s)	12.08 ± 2.46	65
LAYBA-LYM (109/L)	1.76 ± 1.01	147	HV-PT (s)	16.54 ± 14.43	65
LV- LYM (109/L)	0.93 ± 1.41	140	LAYBA-APTT (s)	27.18 ± 5.48	93
HV- LYM (109/L)	1.66 ± 1.93	140	LV-APTT (s)	26.41 ± 4.77	66
LaV- LYM (109/L)	1.34 ± 1.45	151	HV-APTT (s)	33.27 ± 11.81	66
LAYBA-MN (109/L)	0.69 ± 0.32	147	LAYBA-Fibrinogen (g/L)	5.14 ± 1.42	94
OA-MN (109/L)	0.68 ± 0.41	148	LV-Fibrinogen (g/L)	5.43 ± 1.24	66
LV-MN (109/L)	0.38 ± 0.24	140	HV-Fibrinogen (g/L)	6.44 ± 1.1	66
HV-MN (109/L)	0.87 ± 0.46	140	LV-D-dimer (µg/L)	5313.47 ± 10,366.62	15
LAYBA-Platelets (1/mL)	253.84 ± 102.75	147	HV-D-dimer (µg/L)	5397.80 ± 10,339.12	15
LV-Platelets (1/mL)	196.06 ± 82.68	140	FI-BGL (mmol/L)	10.63 ± 5.84	152
HV-Platelets (1/mL)	332.41 ± 158.11	140	FI-Ketones (mmol/L)	1.05 ± 1.71	54
LAYBA-Na (mmol/L)	138.21 ± 3.45	150	Preadmission-RR (1/min)	23.89 ± 8.04	150
LV-Na (mmol/L)	133.49 ± 4.56	142	Preadmission-Saturations (%)	90.5 ± 9.96	142
HV-Na (mmol/L)	140.15 ± 5.77	142	Preadmission-Temperature (°C)	37.47 ± 1.21	149
LAYBA-K (mmol/L)	4.56 ± 0.54	150	Preadmission-SBP (mmHg)	139.27 ± 24.98	150
LV-K (mmol/L)	3.86 ± 0.55	140	Preadmission-DBP (mmHg)	75.56 ± 14.14	150
HV-K (mmol/L)	4.94 ± 0.86	140	Preadmission-Pulse (1/min)	90.65 ± 24.15	150
LAYBA-Urea (mmol/L)	8.85 ± 5.45	150	DM duration (years)	14.15 ± 10.8	145
LV-Urea (mmol/L)	7.75 ± 5.5	142	FI-RR (1/min)	26.26 ± 7.88	152
HV-Urea (mmol/L)	14.51 ± 9.28	142	FI-Saturations (%)	91.94 ± 6.24	152
LAYBA-Creatinine (µmol/L)	140.88 ± 125.46	150	FI-FiO2 (%)	47.16 ± 22.41	103
LV-Creatinine (µmol/L)	142.98 ± 156.87	142	FI-Temperature (°C)	37.1 ± 1.4	152
HV-Creatinine (µmol/L)	223.4 ± 244.63	142	FI-SBP (mmHg)	127.74 ± 31.82	152
eGFR	2.62 ± 1.1	152	FI-Pulse (1/min)	94.47 ± 22.33	152
LAYBA-Bilirubin (µmol/L)	7.55 ± 4.08	149	HR-O2 (%)	55.46 ± 22.85	124
LV-Bilirubin (µmol/L)	6.73 ± 3.93	101	LV-BGL (mmol/L)	4.92 ± 2.34	152
HV-Bilirubin (µmol/L)	12.86 ± 11.02	101	HV-BGL (mmol/L)	16.51 ± 6.65	152
LAYBA-ALT (u/L)	20.37 ± 13.93	142	Average BGL (mmol/L)	9.55 ± 3.02	152
LV-ALT (u/L)	29.38 ± 41.64	68	RBGLR below 3 mmol/L	0.01 ± 0.03	151
HV-ALT (u/L)	76.91 ± 160.6	68	RBGLR 4—10 mmol/L	0.61 ± 0.3	151
LAYBA-TP(g/L)	68.75 ± 7.36	149	RBGLR 10.1—14 mmol/L	0.22 ± 0.19	151
LV-TP (g/L)	59.62 ± 7.51	102	RBGLR 14—21 mmol/L	0.11 ± 0.15	151
HV-TP (g/L)	70.02 ± 6.6	102	RBGLR 21.0—27.8 mmol/L	0.03 ± 0.07	151
LAYBA-ALPO4 (g/L)	99.43 ± 45	149	RBGLR above 27.8 mmol/L	0.01 ± 0.03	151

Note. ALT: alanine transaminase; ALPO4: alkaline phosphatase; APTT: activated partial thromboplastin time; BGL: blood glucose level; BGV: blood gas value; Cl: chloride; CRP: c-reactive protein; DBP: diastolic blood pressure; D-dimer: disseminated intravascular coagulation; DM: diabetes mellitus; eGFR: estimated glomerular filtration rate; FI: first inpatient; FiO2: fraction of inspired oxygen; HbA1c: glycated haemoglobin; HCO3: bicarbonate; HR: highest requirement; HV: highest value; K: potassium; LaV: last value; LAYBA: latest available within one year before admission; LV: lowest value; LYM: lymphocytes; MN: monocytes; Na: sodium; NEUT: neutrophils; O2: oxygen; OA: on admission; pH: potential of hydrogen; PT: prothrombin time; RBGLR: ratio of blood glucose level readings; RR: respiratory rate; SBP: systolic blood pressure; SD: standard deviation; TP: total protein; WCC: white cell count. Note. eGFR > 90 = Stage 1, 60–89 = Stage 2, 30–59 = Stage 3, 15–29 = Stage 4, <15 = Stage 5-Stage CKD.

**Table 3:** The results of the performed SHAP clustering to generate a mortality risk stratification system.

Characteristics	Cluster 1	Cluster 2	Cluster 3	Cluster 4
Count	21	7	10	8
Mortality rate (%)	0.00 ± 0.00	0.43 ± 0.53	0.70 ± 0.48	0.75 ± 0.46
HR-O2	37.31 ± 9.85	37.57 ± 10.83	80.00 ± 0.00	80.00 ± 0.00
FI-FiO2	36.49 ± 10.06	34.03 ± 10.55	78.00 ± 6.32	32.08 ± 6.87
HV-NEUT	6.08 ± 1.97	14.95 ± 3.46	8.99 ± 4.36	7.47 ± 3.32
FI-RR	22.74 ± 4.95	25.57 ± 7.35	31.80 ± 6.36	28.62 ± 6.7
LV-CRP	29.97 ± 21.68	42.67 ± 41.62	75.09 ± 62.95	37.35 ± 33.85
LaV-LYM	1.25 ± 0.5	1.18 ± 0.49	1.10 ± 0.4	0.99 ± 0.62
LAYBA-PT	11.57 ± 0.89	11.36 ± 0.58	12.32 ± 1.87	11.58 ± 0.51
OA-MN	0.72 ± 0.32	0.68 ± 0.3	0.48 ± 0.24	0.63 ± 0.3
LAYBA-NEUT	4.57 ± 1.56	5.19 ± 0.97	5.61 ± 1.73	5.32 ± 1.48
LV-Platelets	189.2 ± 69.29	175 ± 69.63	210.26 ± 70.85	151.43 ± 42.15
LAYBA-MN	0.71 ± 0.25	0.54 ± 0.15	0.64 ± 0.23	0.64 ± 0.13
HV-Platelets	272.1 ± 113.01	420.00 ± 157.79	366.96 ± 152.35	269.68 ± 103.64
FI-BGL	9.67 ± 4.13	13.09 ± 6.42	10.59 ± 4.37	9.85 ± 4.95

Note. BGL: blood glucose level; CRP: c-reactive protein; FI: first inpatient; FiO2: fraction of inspired oxygen; HR: highest requirement; HV: highest value; LaV: last value; LAYBA: latest available within one year before admission; LV: lowest value; LYM: lymphocytes; MN: monocytes; NEUT: neutrophils; OA: on admission; O2: oxygen; PT: prothrombin time; RR: respiratory rate; SD: standard deviation.

**Table 4:** The results of the performed updated SHAP clustering to generate a mortality risk stratification system.

Characteristics	Cluster 1	Cluster 2	Cluster 3	Cluster 4
Count	23	10	7	6
Mortality rate (%)	0.04 ± 0.021	0.50 ± 0.53	0.71 ± 0.49	0.83 ± 0.41
HR-O2	40.07 ± 10.69	41.31 ± 10.70	80 ± 0.00	80 ± 0.00
FI-FiO2	34.07 ± 7.69	33.67 ± 7.16	80.00 ± 0.00	51.60 ± 11.36
HV-NEUT	5.68 ± 1.91	14.59 ± 3.60	9.04 ± 4.09	9.07 ± 4.76
FI-RR	22.78 ± 4.17	26.10 ± 7.29	28.71 ± 6.82	30.17 ± 6.46
LV-CRP	28.83 ± 14.59	38.44 ± 33.25	89.35 ± 62.95	106.84 ± 58.60

Note. FI: first inpatient; FiO2: fraction of inspired oxygen; HR: highest requirement; HV: highest value; LV: lowest value; NEUT: neutrophils; O2: oxygen; RR: respiratory rate; SD: standard deviation.

## Abbreviations

AB: AdaBoost; ALT: alanine transaminase; ALPO4: alkaline phosphatase; APTT: activated partial thromboplastin time; BGL: blood glucose level; BGV: blood gas value; CKD: chronic kidney disease; Cl: chloride; COVID-19: coronavirus disease-2019; CRP: c-reactive protein; CT: computed tomography; CTPA: computed tomography pulmonary angiogram; DBP: diastolic blood pressure; D-dimer: disseminated intravascular coagulation; DM: diabetes mellitus; DNAR: do not attempt resuscitation; eGFR: estimated glomerular filtration rate; FI: first inpatient; FiO2: fraction of inspired oxygen; GB: gradient boosting; HbA1c: glycated haemoglobin; HCO3: bicarbonate; HR: highest requirement; HV: highest value; K: potassium; LaV: last value; LAYBA: latest available within one year before admission; LR: logistic regression; LV: lowest value; LYM: lymphocytes; ML: machine learning; MN: monocytes; Na: sodium; NHS: National health Service; NEUT: neutrophils; O2: oxygen; OA: on admission; PC: presenting complaint; PE: pulmonary embolism; pH: potential of hydrogen; PT: prothrombin time; PVD: peripheral vascular disease; RBGLR: ratio of blood glucose level readings; RPLD: reported pre-existing lung disease; RF: random forest; RR: respiratory rate; SBP: systolic blood pressure; SD: standard deviation; SHAP: SHapley Additive exPlanations; SOB: shortness of breath; SVC: support vector classifier; TP: total protein; VRII: variable rate intravenous insulin infusion; WCC: white cell count.

## Appendix A

In this section, the results of hyperparameter tuning are given. Table A1 represents the outcomes of randomised fine-tuning analysis on hyperparameters for all models in the paper. For each model, a search space is studied for the associated hyperparameters then the random search approach is conducted to select hyperparameter values according to the highest performance on the training set.

**Table A1:** The results of the conducted randomised hyperparameter tuning processes for the classifiers in the article.

Model	Hyperparameter	Search Space	Selected
LR	Regularisation strength	{0, 0.10, 0.20, …, 1}	0.40
	class weight	{0, 1, …, 10}	7
	Maximum number of iterations	{1000, 2000, …, 10,000}	7000
GB	Learning rates	{0.01, 0.02, …, 1}	0.10
	Number of boosting stages	{20, 40, …, 200}	160
	Minimum number of samples required to split an internal node	{1, 2, …, 10}	2
	Minimum number of samples required to be at a leaf node	{{1, 2, …, 10}	6
	Maximum depth of the individual estimators	{1, 2, …, 10}	9
AB	Maximum number of estimators at which boosting is terminated	{10, 20, …, 100}	90
	Learning rates	{0.01, 0.02, …, 1}	1.58
RF	number of trees	{50, 100, …, 500}	20
	Maximum depth of the tree	{1, 2, …, 10}	3
	Minimum number of samples required to split an internal node,	{1, 2, …, 10}	4
	Minimum number of samples required to be at a leaf node	{1, 2, …, 10}	6
	Maximum number of leaf nodes	{1, 2, …, 10}	3
	minimum impurity decrease	{0, 0.001, 0.002, …, 0.010}	0.004
	Cost complexity pruning factor	{0.01, 0.02, …, 0.10}	0.01
	Minimum weighted fraction of the sum total of weights	{0.01, 0.02, …, 0.10}	0.01
SVC	Class weight	{0, 1, …, 10}	6
	Maximum integration	{100, 200, …, 10,000}	2400

Note. AB: AdaBoost; LR: logistic regression; GB: gradient boosting; RF: random forest; SVC: support vector classifier.
